# Prognostic nomogram for overall survival in upper urinary tract urothelial carcinoma (UTUC) patients treated with chemotherapy: a SEER-based retrospective cohort study

**DOI:** 10.1186/s12894-022-01172-8

**Published:** 2023-01-06

**Authors:** Cong Tian, Jun Liu, Lizhe An, Yang Hong, Qingquan Xu

**Affiliations:** 1 Department of Urology, Peking University People’s Hospital, Beijing, 100034 China; 2 Peking University Applied Lithotripsy Institute, Peking University, 133# Fuchengmen Neidajie Street, XiCheng District, Beijing, 100034 China

**Keywords:** SEER, Nomogram, Prognosis, Upper urinary tract urothelial carcinoma, Chemotherapy

## Abstract

**Objective:**

To establish a prognostic nomogram among UTUC patients who received chemotherapy.

**Methods:**

1195 UTUC patients who received chemotherapy were extracted from the Surveillance, Epidemiology, and End Results (SEER) database for the period between 2004 and 2015. Patients were randomly divided into a training and a validation set. Nomogram was constructed to predict 1-, 3-, and 5-year overall survival (OS) in those patients. Receiver-operating characteristic curves (ROCs), calibration plots, and Decision curve analysis (DCA) were applied to assess and compare the discrimination, accuracy, and practicability of the nomogram with 8th American Joint Committee on Cancer (AJCC) tumor node metastasis (TNM) staging system.

**Results:**

Six clinical parameters were identified as independent prognostic factors for UTUC patients’ OS, including age, marital status, TNM stage, and surgical methods of the primary site. The ROC curves showed a satisfactory discrimination capacity of the nomogram, with 1-, 3-, and 5-year area under curve (AUC) values of 0.789, 0.772, and 0.763 in the training set and 0.772, 0.822, and 0.814 in the validation set, respectively. Calibration curves indicated a good agreement between actual observation and nomogram prediction. ROC and DCA curves showed our nomograms exhibited larger benefits than the 8th AJCC-TNM staging system.

**Conclusions:**

A prognostic nomogram was established and validated to present individual predictions of OS among chemotherapeutic UTUC patients. This nomogram may assist clinicians in accurate survival prognostication, treatment decision-making, and design of future clinical trials.

## Introduction

Upper tract urothelial carcinoma (UTUC) is a relatively rare malignancy, including cancer of the renal pelvis and ureter, which accounts for 5% to 10% of all urothelial tumors [1–3]. UTUC is an aggressive tumor with a peak incidence in individuals aged 70–90 years characterized by aggressive growth and variant histology [4]. The high invasiveness of UTUC leads to a poorer prognosis. A study based on the SEER database showed that the 5-year cancer-specific survival (CSS) of UTUC patients was 77% for T2N0 and 39% for lymph node metastasis [5]. Open radical nephroureterectomy (RNU) with bladder cuff excision is the standard treatment of high-risk non-metastatic UTUC. Another option shown to improve survival of patients is peri-operative platinum-based combination chemotherapy [6]. Though large clinical trials are currently lacking to confirm the role of neoadjuvant chemotherapy, it has been shown to result in lower disease recurrence, mortality rates, and an OS and CSS survival benefit compared with RNU alone through several retrospective reviews [7, 8]. In terms of postoperative adjuvant chemotherapy, some scholars have recently confirmed that gemcitabine-platinum combination chemotherapy after nephroureterectomy significantly improved disease-free survival in patients with locally advanced UTUC [9]. For metastatic UTUC patients, systemic chemotherapy (platinum combined chemotherapy) is effective for the first-line treatment of UTUC [6]. Thus far, several scholars have developed corresponding nomograms for UTUC patients to predict patients’ OS and CSS. However, nomograms for patients who received chemotherapy have been lacking. Therefore, an effective prediction model is needed for the accurate assessment of prognosis for these patients, and provide a benchmark for clinical individual decision-making. In this study, we aimed to construct and validate a nomogram for assessing the OS in UTUC patients treated with chemotherapy based on the Surveillance, Epidemiology, and End Results (SEER) database.

## Methods

### Patient and data selection

This study was performed based on the SEER database established by the Department of Cancer Control and Population Sciences of the National Cancer Institute (NCI) in 1973. The database collects data of patients with cancers from 18 districts in the USA, which include clinicopathology, tumor features, and therapeutic details, etc. The database (incidence-seer research plus data, 17 Registries, Nov 2021 Sub (2000–2019)) was used by our study, which covers approximately 26.5% of the U.S. population (based on the 2010 census) and contains one record for each of 8,721,474 tumors. Inclusion criteria were as follows: (1) histologically diagnosed UTUC between 2004 and 2015; 2. Patients who received chemotherapy. The exclusion criteria included the following: 1. Incomplete demographic statistical information such as age, marital status, sex, or race; incomplete clinicopathology information such as tumor-node-metastasis (TNM) stage and pathological grade; incomplete therapeutic information (the interval between diagnosis and treatment, surgical methods of the primary site, or surgery of the regional lymph node); 2. Missing survival status and follow-up information; 3. Diagnostic information from only autopsy or death certificate records; 4. UTUC was not the first primary malignant neoplasm.

SEER * Stat Software (version 8.4.0.1; https://seer.cancer.gov/data-software/) was used to extract information from the SEER database data. 1257 patients with complete clinical data were enrolled in the study, all of which matched the inclusion and exclusion criteria. 2 patients whose survival time was 0 and 60 patients who underwent Multivisceral resection were excluded from the final nomogram. 1195 patients were included in the final model construction. The TNM staging system was reclassified according to version 8 criteria [10].

### Statistical analysis

The primary outcome of the study was OS in UTUC patients who received chemotherapy. OS was defined as the interval between the date of cancer diagnosis and the date of death recorded in the registry. 1195 UTUC patients were randomly assigned to the training and validation sets with a ratio of 7:3. The baseline characteristics between the two groups were analyzed by chi-square test and t-test. The training set was used to develop the original nomogram, while the training set and validation set were both used to draw the receiver operating characteristic (ROC) curve, calibration plots, and decision curve analysis (DCA). Univariate and multivariate Cox proportional hazards regression model were applied to determine independent prognostic factors. Then the nomogram was generated based on the independent prognostic factors calculated by the multivariate Cox model. ROC, AUC, and Harrell’s concordance index (C-index) were used to distinguish the models and compare the prediction probabilities of nomogram and 8th AJCC-TNM staging system in 1-year, 3-year, and 5-year OS. Calibration plots were generated to validate the model by comparing the predicted values and actual observations. DCA was applied to quantify clinical utility and compare prognosis predictive capacity between the nomogram and the 8th AJCC-TNM staging system. All tests were performed using R software (version 4.2.1, http://www.R-project.org). Two-sided *P* < 0.05 was considered statistically significant.

## Results

### Patient baseline characteristics

**Fig. 1:**
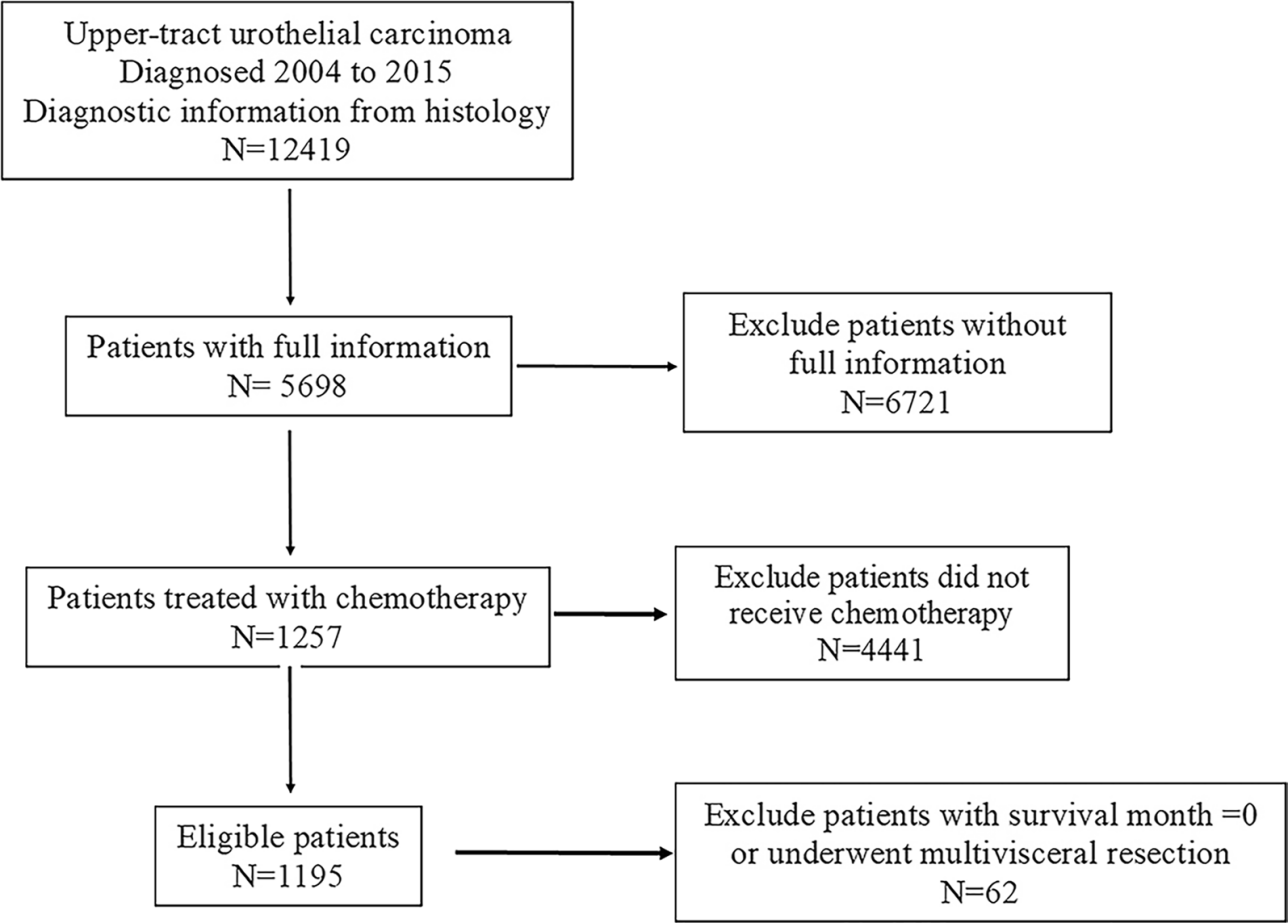
Flowchart of screening process

Overall, 5698 UTUC patients were identified from the SEER database, of which 1257 UTUC patients received chemotherapy. 2 patients whose survival time was 0 and 60 patients who underwent Multivisceral resection were excluded. 1195 patients were further assigned to training and validation sets in a 7:3 ratio. Figure 1 shows the screening process. The demographics, clinicopathological characteristics, and therapeutic information of UTUC patients with or without chemotherapy are presented in Table 1. Details of the training and validation sets are provided in Table 2. For UTUC patients who received chemotherapy, the patients were relatively younger (67.2 years vs 72.1 years, *P* < 0.001) and had a higher grade and TNM stage (*P* < 0.001) compared with those who did not. The surgery of regional lymph nodes was more common among patients who received chemotherapy (*P* < 0.001). There were no statistically significant differences in gender (*P* = 0.076), primary site (*P* = 0.358), and radiotherapy (*P* = 1.000). The baseline features between the training and validation sets were well balanced, as shown in Table 2.

**Table 1:** Demographics, clinicopathologic characteristics, and therapeutic information of the enrolled UTUC patients

Characteristics	Received chemotherapy	Unreceived chemotherapy	Total	*P* value
	(N = 4441)	(N = 1257)	(N = 5698)	
Age (years)				< 0.001*
Mean (SD)	72.1 (11.0)	67.2 (10.4)	71.1 (11.1)	
Sex				0.076
Female	1943 (43.8%)	514 (40.9%)	2457 (43.1%)	
Male	2498 (56.2%)	743 (59.1%)	3241 (56.9%)	
Race				< 0.001*
White	3887 (87.5%)	1051 (83.6%)	4938 (86.7%)	
Black	185 (4.2%)	53 (4.2%)	238 (4.2%)	
Other	369 (8.3%)	153 (12.2%)	522 (9.2%)	
Marital status				< 0.001*
Married	2658 (59.9%)	890 (70.8%)	3548 (62.3%)	
Divorced/widowed/Separated	1363 (30.7%)	248 (19.7%)	1611 (28.3%)	
Unmarried/Single	420 (9.5%)	119 (9.5%)	539 (9.5%)	
Primary Site				0.358
Renal Pelvis	2933 (66.0%)	812 (64.6%)	3745 (65.7%)	
Ureter	1508 (34.0%)	445 (35.4%)	1953 (34.3%)	
Grade				< 0.001*
Grade I	246 (5.5%)	21 (1.7%)	267 (4.7%)	
Grade II	801 (18.0%)	77 (6.1%)	878 (15.4%)	
Grade III	1253 (28.2%)	404 (32.1%)	1657 (29.1%)	
Grade IV	2141 (48.2%)	755 (60.1%)	2896 (50.8%)	
T Stage				< 0.001*
T1	1642 (37.0%)	177 (14.1%)	1819 (31.9%)	
T2	881 (19.8%)	132 (10.5%)	1013 (17.8%)	
T3	1598 (36.0%)	694 (55.2%)	2292 (40.2%)	
T4	320 (7.2%)	254 (20.2%)	574 (10.1%)	
N Stage				< 0.001*
N0	4123 (92.8%)	779 (62.0%)	4902 (86.0%)	
N1	186 (4.2%)	262 (20.8%)	448 (7.9%)	
N2	132 (3.0%)	216 (17.2%)	348 (6.1%)	
M Stage				< 0.001*
M0	4286 (96.5%)	1024 (81.5%)	5310 (93.2%)	
M1	155 (3.5%)	233 (18.5%)	388 (6.8%)	
Radiation				1.000
No/Unknown	19 (0.4%)	6 (0.5%)	25 (0.4%)	
Yes	4422 (99.6%)	1251 (99.5%)	5673 (99.6%)	
Surgical methods of the primary site				< 0.001*
No	26 (0.6%)	124 (9.9%)	150 (2.6%)	
Local Mass resection/destruction	163 (3.7%)	49 (3.9%)	212 (3.7%)	
Partial nephrectomy/ureterectomy	472 (10.6%)	114 (9.1%)	586 (10.3%)	
Radical nephroureterectomy	2600 (58.5%)	586 (46.6%)	3186 (55.9%)	
Radical nephrectomy	1014 (22.8%)	324 (25.8%)	1338 (23.5%)	
Multivisceral resection	166 (3.7%)	60 (4.8%)	226 (4.0%)	
Surgery of Lymph nodes (LNs)				< 0.001*
No	3413 (76.9%)	716 (57.0%)	4129 (72.5%)	
1 to 3 regional LNs removed	610 (13.7%)	268 (21.3%)	878 (15.4%)	
4 or more regional LNs removed	418 (9.4%)	273 (21.7%)	691 (12.1%)	

*P < 0.05 indicating statistical significance

**Table 2:** Demographics, clinicopathologic characteristics, and therapeutic information of the training set and validation set

Characteristics	Training set	Validation set	Overall	*P* value
	(N = 836)	(N = 359)		
Age (years)				0.268
Mean (SD)	67.0 (10.2)	67.8 (10.6)	67.3 (10.4)	
Sex				0.554
Female	343 (41.0%)	140 (39.0%)	483 (40.4%)	
Male	493 (59.0%)	219 (61.0%)	712 (59.6%)	
Race				0.151
White	690 (82.5%)	307 (85.5%)	997 (83.4%)	
Black	41 (4.9%)	9 (2.5%)	50 (4.2%)	
Other	105 (12.6%)	43 (12.0%)	148 (12.4%)	
Marital status				0.536
Married	587 (70.2%)	263 (73.3%)	850 (71.1%)	
Divorced/widowed/Separated	169 (20.2%)	67 (18.7%)	236 (19.7%)	
Unmarried/Single	80 (9.6%)	29 (8.1%)	109 (9.1%)	
Primary Site				0.422
Renal Pelvis	537 (64.2%)	240 (66.9%)	777 (65.0%)	
Ureter	299 (35.8%)	119 (33.1%)	418 (35.0%)	
Grade				0.484
Grade I	16 (1.9%)	3 (0.8%)	19 (1.6%)	
Grade II	48 (5.7%)	25 (7.0%)	73 (6.1%)	
Grade III	266 (31.8%)	114 (31.8%)	380 (31.8%)	
Grade IV	506 (60.5%)	217 (60.4%)	723 (60.5%)	
T Stage				0.457
T1	127 (15.2%)	44 (12.3%)	171 (14.3%)	
T2	90 (10.8%)	38 (10.6%)	128 (10.7%)	
T3	466 (55.7%)	201 (56.0%)	667 (55.8%)	
T4	153 (18.3%)	76 (21.2%)	229 (19.2%)	
N Stage				0.977
N0	520 (62.2%)	221 (61.6%)	741 (62.0%)	
N1	171 (20.5%)	75 (20.9%)	246 (20.6%)	
N2	145 (17.3%)	63 (17.5%)	208 (17.4%)	
M Stage				0.385
M0	690 (82.5%)	288 (80.2%)	978 (81.8%)	
M1	146 (17.5%)	71 (19.8%)	217 (18.2%)	
Radiation				0.329
No/Unknown	2 (0.2%)	3 (0.8%)	5 (0.4%)	
Yes	834 (99.8%)	356 (99.2%)	1190(99.6%)	
Surgical methods of the primary site				0.669
No	90 (10.8%)	32 (8.9%)	122 (10.2%)	
Local Mass resection/destruction	31 (3.7%)	18 (5.0%)	49 (4.1%)	
Partial nephrectomy/ureterectomy	79 (9.4%)	35 (9.7%)	114 (9.5%)	
Radical nephroureterectomy	414 (49.5%)	172 (47.9%)	586 (49.0%)	
Radical nephrectomy	222 (26.6%)	102 (28.4%)	324 (27.1%)	
Surgery of Lymph nodes (LNs)				0.925
No	484 (57.9%)	207 (57.7%)	691 (57.8%)	
1 to 3 regional LNs removed	174 (20.8%)	78 (21.7%)	252 (21.1%)	
4 or more regional LNs removed	178 (21.3%)	74 (20.6%)	252 (21.1%)	

*P < 0.05 indicating statistical significance

### Univariate and multivariate analyses

The following variables were included in the univariate cox regression analysis: race, age, gender, marital status, TNM stage, tumor grade, surgical methods of the primary site, radiotherapy, and surgery of regional lymph nodes. Based on the results of the univariate analysis, variables that *P* < 0.05 were included in the multivariate analysis, including age, marital status, TNM stage, and surgical methods of the primary site. Multivariate Cox regression analysis further confirmed that age, TNM stage, marital status, and surgical methods of the primary site were independent prognostic factors for OS. The results of univariate and multivariate Cox regression analysis are shown in Table 3.

**Table 3:** Univariate and multivariate COX regression analysis for OS in the training sets

	Univariate analysis			Multivariate analysis		
	HR	95%CI	*P* value	HR	95%CI	*P* value
Age	1.028	1.02–1.037	< 0.001*	1.027	1.018–1.036	< 0.001*
Sex						
Female	Ref					
Male	0.949	0.805–1.119	0.532			
Race						
White	Ref					
Black	0.848	0.579–1.241	0.396			
Other	1.050	0.823–1.339	0.696			
Marital status						
Married	Ref					
Divorced/widowed/Separated	1.269	1.038–1.549	0.020*	1.114	0.907–1.367	0.302
Unmarried/Single	1.282	0.975–1.685	0.076	1.374	1.039–1.816	0.026*
Primary Site						
Renal Pelvis	Ref					
Ureter	1.003	0.848–1.186	0.973			
Grade						
Grade I	Ref					
Grade II	1.101	0.545–2.224	0.788			
Grade III	1.409	0.746–2.658	0.290			
Grade IV	1.161	0.618–2.177	0.642			
T Stage						
T1	Ref					
T2	0.826	0.581–1.172	0.284	1.064	0.744–1.519	0.735
T3	1.084	0.847–1.387	0.520	1.349	1.044–1.743	0.022*
T4	2.405	1.819–3.180	< 0.001*	2.309	1.721–3.097	< 0.001*
N Stage						
N0	Ref					
N1	1.880	1.542–2.292	< 0.001*	1.284	1.035–1.593	0.023*
N2	2.013	1.633–2.482	< 0.001*	1.354	1.082–1.695	0.008*
M Stage						
M0	Ref					
M1	3.172	2.609–3.857	< 0.001*	1.985	1.579–2.495	< 0.001*
Radiation						
No/Unknown	Ref					
Yes	1.241	0.174–8.83	0.829			
Surgical methods of the primary site						
No	Ref					
Local Mass resection/destruction	0.442	0.287–0.679	< 0.001*	0.647	0.412–1.016	0.059
Partial nephrectomy/ureterectomy	0.224	0.156–0.320	< 0.001*	0.445	0.301–0.660	< 0.001*
Radical nephroureterectomy	0.221	0.171–0.284	< 0.001*	0.380	0.283–0.509	< 0.001*
Radical nephrectomy	06.35	0.273–0.464	< 0.001*	0.485	0.364–0.646	< 0.001*
Surgery of Lymph nodes (LNs)						
No	Ref					
1 to 3 regional LNs removed	0.994	0.810–1.219	0.957			
4 or more regional LNs removed	0.830	0.672–1.025	0.083			

*P < 0.05 indicating statistical significance

### Nomogram construction and validation

**Fig. 2:**
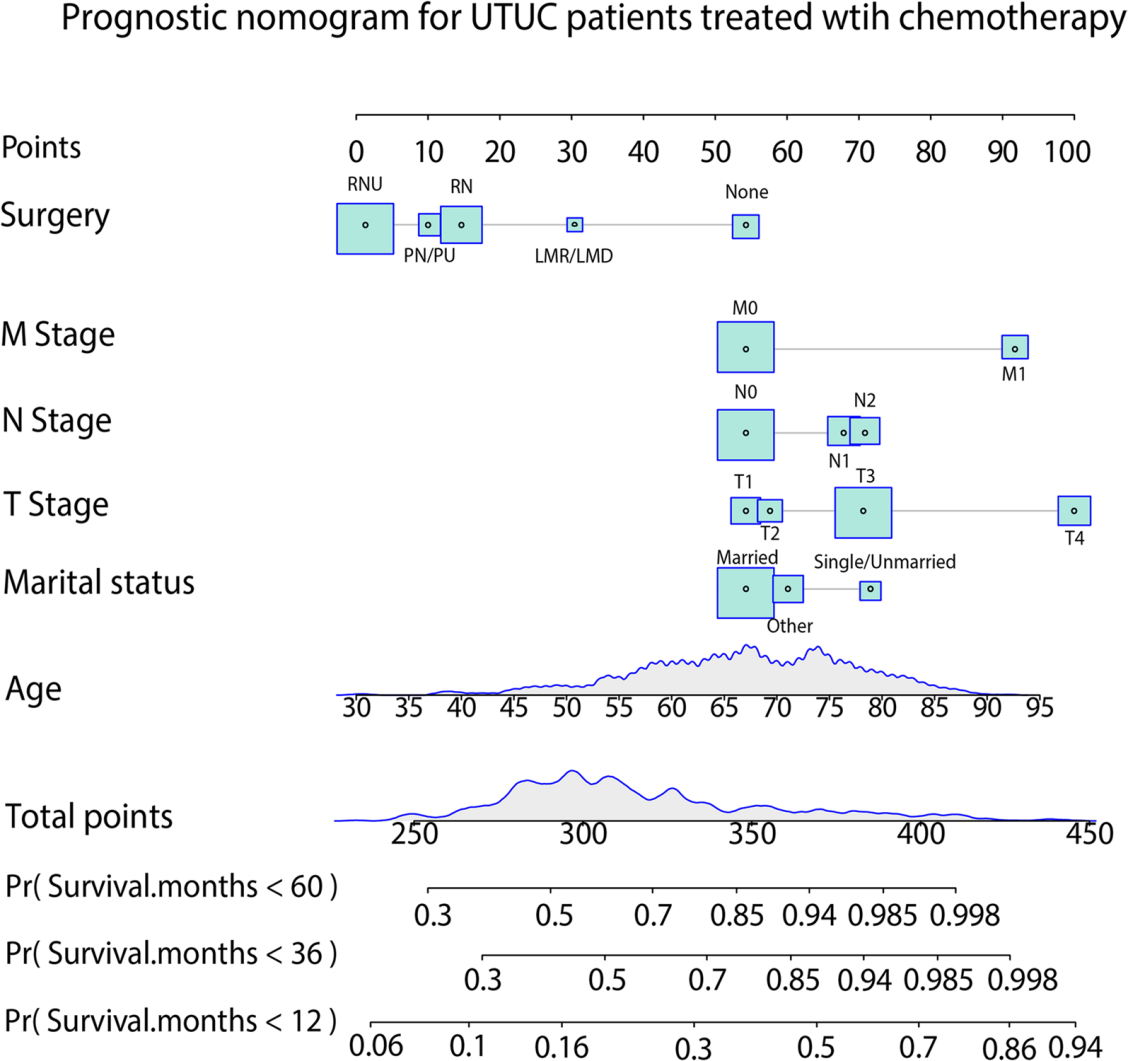
Nomogram predicting 1-, 3-, and 5-year overall survival for UTUC patients treated with chemotherapy. Nomograms were built by using data from the training sets. RNU: Radical nephroureterectomy; PN/PU: Partial nephrectomy/ureterectomy; RN: Radical nephrectomy; LMR/LMD: Local Mass resection/destruction

Based on multivariate Cox regression analysis, our nomogram was developed to predict OS in UTUC patients who received chemotherapy. In the nomogram depicted in Fig. 2, the score of each variable was calculated by applying a ranking scale drawn by the intersection of the vertical line from each independent factor to the point axis. The 1-, 3-, and 5-year survival probabilities can then be further obtained by summing the scores.

**Fig. 3:**
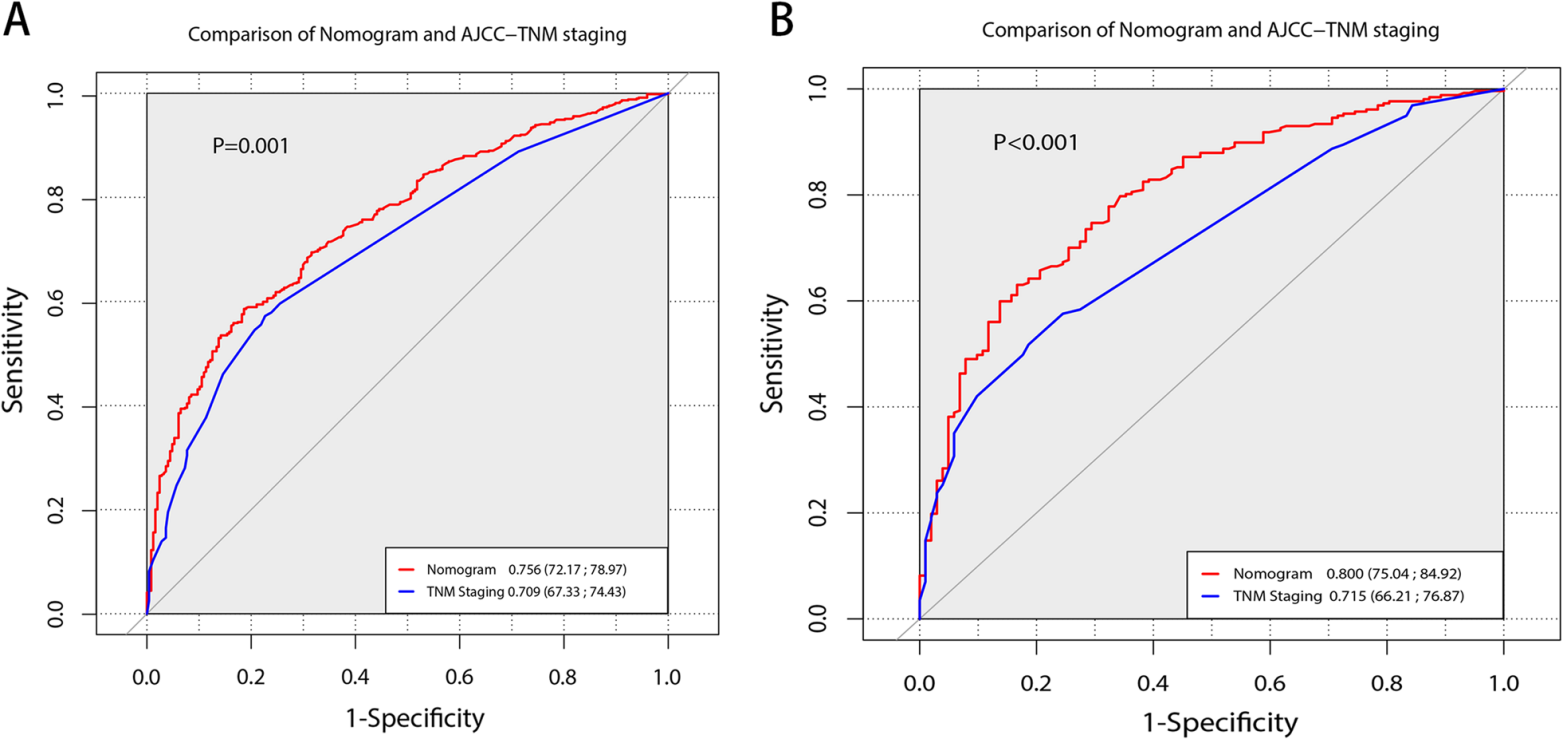
ROC curves of the nomogram for predicting OS. The figure shows AUC of ROC in patients of training (**A**) and validation set (**B**)

**Fig. 4:**
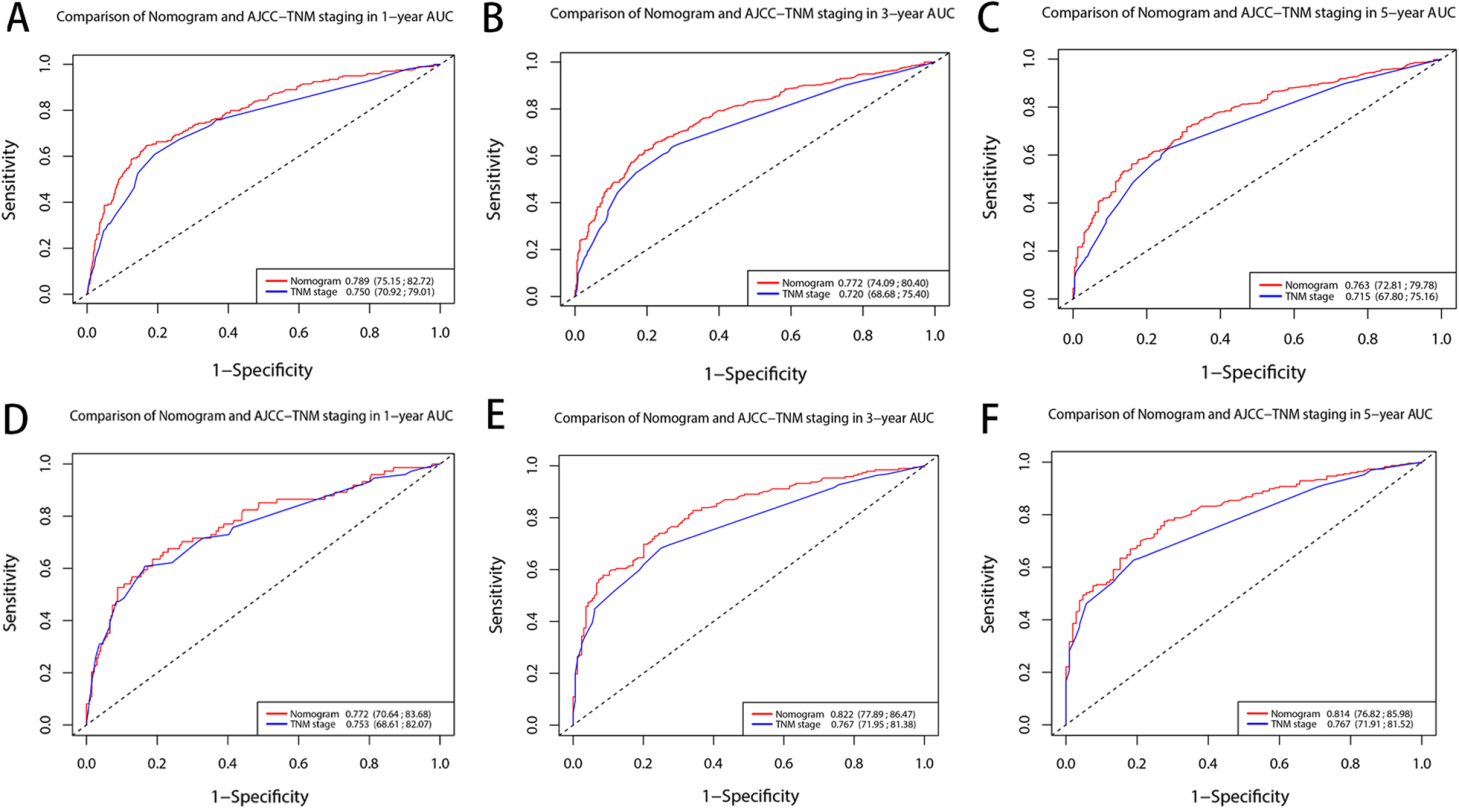
ROC curves of the nomogram for OS compared with TNM staging. The figure shows AUC of ROC for 1 (**A**), 3 (**B**), and 5 years (**C**) in the training set and 1 (**D**), 3 (**E**), and 5 years (**F**) in the validation set

**Fig. 5:**
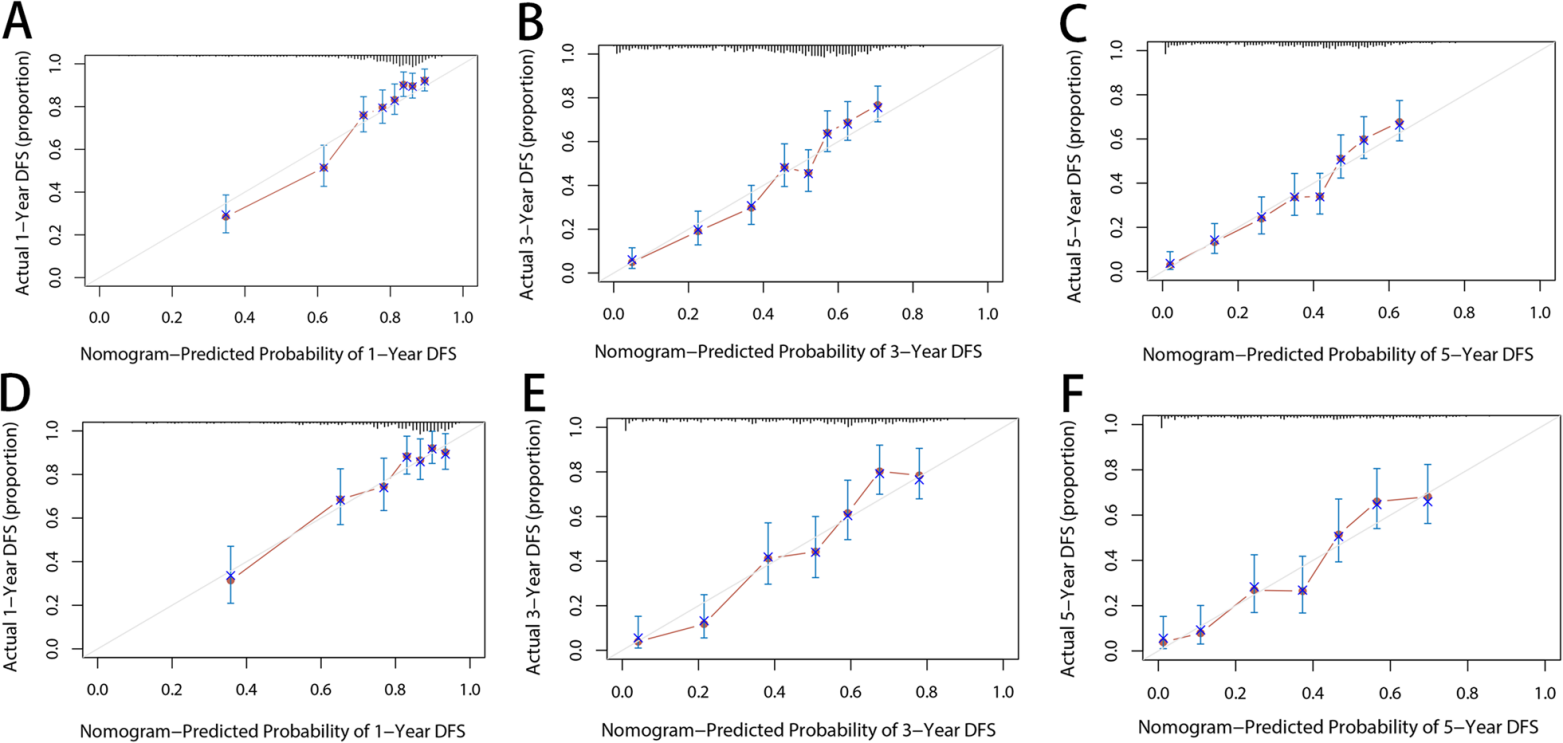
Calibration plots for the nomogram. The figure shows calibration capacity for 1 (**A**), 3 (**B**), and 5 years (**C**) in the training set; 1 (**D**), 3 (**E**), and 5 years (**F**) in the validation set

**Fig. 6:**
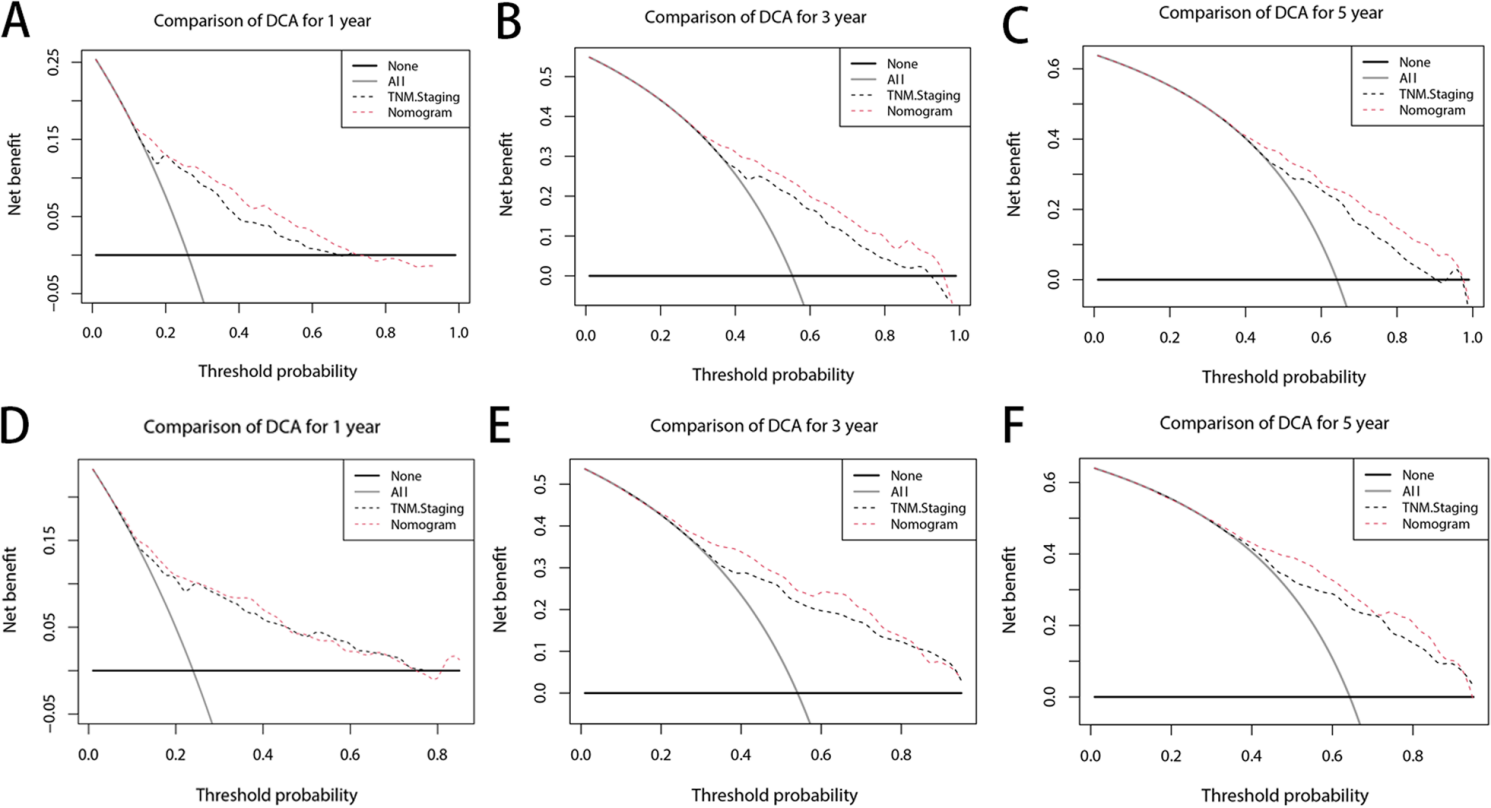
Comparison of DCA between the nomogram and 8th AJCC-TNM staging system for 1 (**A**), 3 (**B**), and 5 years (**C**) in the training set; 1 (**D**), 3 (**E**), and 5 years (**F**) in the validation set

The C index of our model is significantly higher than that of the TNM staging system (0.7108 VS 0.6723, *P* < 0.001). As shown in Figs. 3 and 4, our nomogram shows the satisfactory discriminative capacity of OS prediction. The predictive accuracy of our nomogram was superior to that of the AJCC-TNM staging system (*P* = 0.001 for the training set, *P* < 0.001 for the validation set). The area under curve (AUC) was 0.789 (1-year), 0.772 (3-year) and 0.763 (5-year) for the training set and 0.772 (1-year), 0.822 (3-year) and 0.814 (5-year) for the validation set, respectively. The calibration plot was used to reflect the agreement between the nomogram prediction and the actual observation of the patients’ OS. The calibration plots indicated good calibration (Fig. 5). Figure 6 shows the comparison of DCA between our nomogram and AJCC-TNM staging system.

## Discussion

Nowadays, the AJCC-TNM staging system is a common tool for predicting OS in UTUC patients [6, 11]. However, the TNM staging system only involves the local progression and distant metastasis of the tumor, which leads to a great difference in the final prognosis. A major benefit of a nomogram is that it can provide prognostic values to predict the patient's prognosis more accurately [12]. In our study, data were extracted from the SEER database to establish a prognostic nomogram for UTUC patients who received chemotherapy. To our knowledge, this is the first attempt to establish a predictive model for this subgroup.

Our study showed that patients who received chemotherapy had higher grade and TNM stage than patients who did not. This is consistent with the founding of other scholars [13]. The recent EAU guideline recommends chemotherapy for patients with high-risk non-metastatic UTUC and metastatic UTUC patients [6]. Goldberg, H et al. stated that perioperative chemotherapy had no effect on cancer specific mortality in high-risk non-metastatic patients through SEER database [13]. However, Zhai, T. S. et al. found that the beneficial effect of perioperative chemotherapy on OS was to be evident in pT3/pT4 and pN + patients but to reduce cancer-specific survival for pT1 and OS for pT2 patients [14]. For low-risk UTUC patients, the benefits of perioperative chemotherapy need to be carefully weighed against the risk. Wang, M et al. found that patients who receive chemotherapy was associated with improved overall all survival [15]. All these conclusions need to be interpreted with caution because of the selection bias, residual unmeasured confounding, and lack of timing, protocol, tolerability, and complications of chemotherapy. We still need the large randomized controlled study to access the effects of neoadjuvant chemotherapy and adjuvant chemotherapy, respectively. Clinically, chemotherapy should be individualized for UTUC patients. Except for the grade, stage, or other characteristics of the tumor, the patient's age, health condition, and renal function should also be considered, especially for postoperative patients [16]. Therefore, these patients belong to a special group, and targeted nomograms should be applied to access the prognosis of patients and aid clinicians to make decisions.

Our research indicated that age, TNM stage, marital status, and surgical methods of the primary site were independent prognostic factors for OS of UTUC patients treated with chemotherapy. Our nomogram was developed based on these prognostic factors to predict OS at 1, 3, and 5 years. The nomogram shows good prognostic ability and reliability. ROC and DCA curves showed our nomograms exhibited larger benefits than the 8th AJCC-TNM staging system. The results were consistent in both the training set and the internal validation set. However, Wang, M. suggested the use of AJCC TNM staging may better guide clinical decisions when predicting prognosis in high-grade patients [15]. The full information such as comorbidity are inaccessible from the SEER database, and selection bias could not be avoided in this research. External validation is still needed.

Several nomograms have been established to predict the prognosis of UTUC patients. Wu J et al. established a nomogram based on the SEER database, in which the gender, age, marital status, histology, seer stage, grade, surgery, radiotherapy, and chemotherapy were identified as prognostic factors for patients’ OS [17], Qi F et al. did a similar study, and the model also included radiotherapy [18]. At present, whether radiotherapy can improve the prognosis of UTUC patients is controversial and the combined effect of chemotherapy and radiotherapy remains questionable [6, 19]. In our study, radiotherapy did not improve patients' OS, which needs to be confirmed by further studies. In another study conducted by Li Z et al., three independent factors were identified for patients with invasive UTUC: age, TNM stage, and grade. The nomogram indicated better predictive accuracy than the AJCC-TNM staging system [20], Li C et al. constructed a nomogram by using the competing risk model. They found that LNP and LNR were associated with the CSD of UTUC patients [21]. Previous models mainly focused on the whole UTUC population to construct corresponding models, while our study focused on the special group that received chemotherapy. This is one of the strengths of our study over previous studies.

One further strength lies in the following aspect. Surgical methods were incorporated into our nomogram for predicting the OS of UTUC patients. We built a nomogram based on age, marital status, TNM stage, and surgical methods of the primary site to predict the OS of UTUC patients who received chemotherapy. Surgical methods of the primary site were as follows: local Mass resection/destruction, radical nephroureterectomy, partial nephrectomy/ureterectomy, and radical nephrectomy. Open radical nephroureterectomy with bladder cuff excision is the standard treatment for high-risk non-metastatic UTUC [6]. However, in real clinical practice, the surgical methods need to be combined with the actual situation of the patient. In some cases, such as solitary kidney, severely impaired renal function, or the patient cannot tolerate general anesthesia, Kidney-sparing surgery combined with postoperative chemotherapy is also a kind of choice, so our model was in line with the real clinical condition. The prognostic nomogram that we established displayed a better prognosis prediction capacity compared to the 8th AJCC-TNM staging system. Therefore, for UTUC patients treated with chemotherapy, our nomogram may assist clinicians in accurate survival prognostication, treatment decision-making, and design of future clinical trials. At present, radical nephroureterectomy also has a lot of innovation such as laparoscopic radical nephroureterectomy with only three trocars [22] and these various new techniques can be incorporated into the futural clinical nomogram.

UTUC with histological variants were excluded from our study such as squamous and sarcomatoid variants. Histological variants of urothelial carcinoma are relatively rare, with approximately 25% of UTUC containing variant histology. Variant histology was associated with higher grades and poorer oncological outcomes [23, 24]. The validity of chemotherapy for this subgroup is controversial. A retrospective study demonstrated that the improvement in OS of these patients was not statistically significant [25]. Therefore, these patients were excluded from our study, thereby improving the accuracy of the study.

Our study had certain drawbacks. First, our study was a retrospective research design and selection bias inevitably existed. Second, the SEER database is short of detailed information about specific chemotherapy regimens, comorbidity and renal function. The latter emerges as an important factor influencing whether patients receive chemotherapy and its efficacy. Third, our nomograms should be externally validated for prediction capacity by large cohorts. Moreover, since more that 10% of UTUC patients had concomitant bladder cancer [26], and some patients presented with recurrence in the bladder cancer following treatment [27]. This study did not consider concurrent or heterochronous bladder cancer. Further study is required to address this issue.

## Conclusion

At present, no suitable model exists to predict OS in UTUC patients treated with chemotherapy. The prognostic predictive capacity and reliability of our model were acceptable. Our model may provide meaningful reference to assist clinicians in accurate survival prognostication, treatment decision-making, and design of future clinical trials.

## Data Availability

The datasets analysed during the current study can be obtained by the SEER*Stat software (https://seer.cancer.gov/). All data are fully available without restriction. The author obtained access to the SEER database (Accession Number: 14467-Nov2021).
