# Correction to: Prognostic nomogram for overall survival in upper urinary tract urothelial carcinoma (UTUC) patients treated with chemotherapy: a SEER-based retrospective cohort study

**DOI:** 10.1186/s12894-023-01231-8

**Published:** 2023-06-24

**Authors:** Cong Tian, Jun Liu, Lizhe An, Yang Hong, Qingquan Xu

**Affiliations:** 1 Department of Urology, Peking University People’s Hospital, Beijing, 100034 China; 2 Peking University Applied Lithotripsy Institute, Peking University, 133# Fuchengmen Neidajie Street, XiCheng District, Beijing, 100034 China

**Correction to: BMC Urology (2023) 23:1**

10.1186/s12894-022-01172-8

Following publication of the original article [1], In table 1, First line, second column, “Received chemotherapy” were supposed to be “Unreceived chemotherapy”. First line, third column, “Unreceived chemotherapy” were supposed to be “Received chemotherapy”. 

The original article has been corrected.

**Table 1:** Demographics, clinicopathologic characteristics, and therapeutic information of the enrolled UTUC patients.

Characteristics	Unreceived chemotherapy	Received chemotherapy	Total	P value
Number of patients (%) Age (years)	4441 (77.9%)	1257 (22.1%)	5698 (100%)	< 0.001*
Mean (SD)	72.1 (11.0)	67.2 (10.4)	71.1 (11.1)	
Sex				0.076
Female	1943 (43.8%)	514 (40.9%)	2457 (43.1%)	
Male	2498 (56.2%)	743 (59.1%)	3241 (56.9%)	
Race				< 0.001*
White	3887 (87.5%)	1051 (83.6%)	4938 (86.7%)	
Black	185 (4.2%)	53 (4.2%)	238 (4.2%)	
Other	369 (8.3%)	153 (12.2%)	522 (9.2%)	
Marital status				< 0.001*
Married	2658 (59.9%)	890 (70.8%)	3548 (62.3%)	
Divorced/widowed/Separated	1363 (30.7%)	248 (19.7%)	1611 (28.3%)	
Unmarried/Single	420 (9.5%)	119 (9.5%)	539 (9.5%)	
Primary Site				0.358
Renal Pelvis	2933 (66.0%)	812 (64.6%)	3745 (65.7%)	
Ureter	1508 (34.0%)	445 (35.4%)	1953 (34.3%)	
Grade				< 0.001*
Grade I	246 (5.5%)	21 (1.7%)	267 (4.7%)	
Grade II	801 (18.0%)	77 (6.1%)	878 (15.4%)	
Grade III	1253 (28.2%)	404 (32.1%)	1657 (29.1%)	
Grade IV	2141 (48.2%)	755 (60.1%)	2896 (50.8%)	
T Stage				< 0.001*
T1	1642 (37.0%)	177 (14.1%)	1819 (31.9%)	
T2	881 (19.8%)	132 (10.5%)	1013 (17.8%)	
T3	1598 (36.0%)	694 (55.2%)	2292 (40.2%)	
T4	320 (7.2%)	254 (20.2%)	574 (10.1%)	
N Stage				< 0.001*
N0	4123 (92.8%)	779 (62.0%)	4902 (86.0%)	
N1	186 (4.2%)	262 (20.8%)	448 (7.9%)	
N2	132 (3.0%)	216 (17.2%)	348 (6.1%)	
M Stage				< 0.001*
M0	4286 (96.5%)	1024 (81.5%)	5310 (93.2%)	
M1	155 (3.5%)	233 (18.5%)	388 (6.8%)	
Radiation				1.000
No/Unknown	19 (0.4%)	6 (0.5%)	25 (0.4%)	
Yes	4422 (99.6%)	1251 (99.5%)	5673 (99.6%)	
Surgical methods of the primary site				< 0.001*
No	26 (0.6%)	124 (9.9%)	150 (2.6%)	
Local Mass resection/destruction	163 (3.7%)	49 (3.9%)	212 (3.7%)	
Partial nephrectomy/ureterectomy	472 (10.6%)	114 (9.1%)	586 (10.3%)	
Radical nephroureterectomy	2600 (58.5%)	586 (46.6%)	3186 (55.9%)	
Radical nephrectomy	1014 (22.8%)	324 (25.8%)	1338 (23.5%)	
Multivisceral resection	166 (3.7%)	60 (4.8%)	226 (4.0%)	
Surgery of Lymph nodes (LNs)				< 0.001*
No	3413 (76.9%)	716 (57.0%)	4129 (72.5%)	
1 to 3 regional LNs removed	610 (13.7%)	268 (21.3%)	878 (15.4%)	
4 or more regional LNs removed	418 (9.4%)	273 (21.7%)	691 (12.1%)	

*P < 0.05 indicating statistical significance.
